# Long-term outcomes and toxicity of radiotherapy for WHO Grade II and III meningiomas: a retrospective analysis of 98 patients

**DOI:** 10.1007/s11060-025-05255-y

**Published:** 2025-10-24

**Authors:** Kerem Tuna Tas, Finja Svende Höft, Linda Agolli, Tasneim Abdelrahman Mohamed, Fatima Frosan Sheikhzadeh, Philipp Lishewski, Markus Schymalla, Klemens Zink, Hilke Vorwerk, Semi B. Harrabi, Daniel Habermehl, Sebastian Adeberg, Ahmed Gawish

**Affiliations:** 1 Department of Radiotherapy and Radiation Oncology, Philipps-Universität Marburg, Marburg, Germany; 2 Department of Radiotherapy and Radiation Oncology, Marburg University Hospital, Marburg, Deutschland; 3 Marburg Ion-Beam Therapy Center (MIT), Department of Radiotherapy and Radiation Oncology, Marburg University Hospital, Marburg, Germany; 4 University Cancer Center (UCT) Frankfurt – Marburg, Marburg, Germany; 5 Department of Radiotherapy, Giessen University Hospital, Justus Liebig University, Giessen, Germany; 6 LOEWE Research Cluster for Advanced Medical Physics in Imaging and Therapy, (ADMIT), TH Mittel Hessen University of Applied Sciences, Giessen, Germany; 7 Department of Radiation Oncology, Heidelberg University Hospital, Heidelberg, Germany; 8 Heidelberg Ion-Beam Therapy Center (HIT), Heidelberg, Germany

**Keywords:** Stereotactic radiosurgery, Radiation oncology, Meningioma

## Abstract

**Background:**

This retrospective study evaluated outcomes and toxicity in patients with WHO Grade II and III meningiomas treated with modern photon or particle radiotherapy, with emphasis on skull base versus non-skull base tumors.

**Methods:**

Ninety-seven patients received photon (58.2%) or particle therapy (41.8%). Median age was 61 years (range, 15–88). Tumor location was non-skull base in 69.1% and skull base in 30.9%. All patients underwent fractionated radiotherapy with a median dose of 59.4 Gy (range, 34–68). Follow-up included MRI and assessment of local control (LC), progression-free survival (PFS), overall survival (OS), and toxicity (CTCAE v5).

**Results:**

At last follow-up, 94 patients (96.9%) were alive. Median OS was not reached, with survival rates of 100% at 2 and 5 years and 99% at 8 years. Median PFS was 32.0 months, with 2- and 5-year rates of 88.7% and 66.0%. Median LC was 33.0 months, with 2- and 5-year rates of 91.8% and 72.2%. Disease progression occurred in 40 patients (41.2%), including 30 in-field and 22 outside the irradiated volume. Early toxicities were mainly Grade I–II, most commonly alopecia, fatigue, and headache. Late toxicities were less frequent, including headache, seizures, vertigo, and radiation-induced cerebral contrast enhancement (RICE). Severe late toxicity (Grade III) was rare (*n* = 3). Particle therapy was associated with lower rates of vertigo and headache.

**Conclusion:**

High-precision photon and particle radiotherapy achieved effective long-term control with favorable safety in high-grade meningiomas. Most adverse effects were mild and manageable, supporting the role of particle therapy in reducing selected late toxicities.

## Introduction

Meningiomas are the most common primary tumors of the central nervous system, representing 30–37% of all intracranial neoplasms [1, 2]. While the majority are benign (WHO Grade I), a significant minority are classified as WHO Grade II (atypical) and Grade III (anaplastic) and are distinguished by their aggressive clinical behavior, higher recurrence rates, and reduced long-term survival compared to lower-grade lesions [3, 4].

Epidemiologically, WHO Grade II and III meningiomas comprise 4–5% and approximately 1–2% of all meningiomas, respectively, and exhibit a slight male predominance, in contrast to lower-grade tumors which are more common in females [2, 5]. These high-grade lesions are found less frequently at the cranial base compared to other intracranial locations [6]. Incidence increases with age, and certain population groups, such as Black individuals, demonstrate a higher occurrence rate [1, 2].

The cornerstone of management is maximal safe surgical resection. However, complete excision is often unfeasible for tumors near critical neurovascular structures or at the skull base, resulting in substantial recurrence rates. For WHO Grade II meningiomas, five-year recurrence can approach 29–52%, while Grade III tumors recur at rates up to 95% even after gross total resection [3, 4, 7, 8]. Recurrences are particularly frequent with incomplete resection or unfavorable histology, leading to significantly poorer outcomes.

Radiotherapy (RT) has become an integral component in the multidisciplinary management of WHO Grade II and III meningiomas, especially when surgery is incomplete or the risk of recurrence is high. Multiple studies support the use of adjuvant RT for both residual disease and after complete resection in high-grade cases, demonstrating reduced recurrence and improved progression-free survival when modern photon or particle-based RT is applied [4, 9–11]. Advances in RT delivery, including intensity-modulated radiotherapy (IMRT), fractionated stereotactic techniques, and proton or carbon ion therapy, have enabled more precise targeting of tumor volumes while minimizing collateral damage to healthy tissue, thus optimizing outcomes [9, 11, 12, 18].

In line with this, El Shafie et al. reported excellent long-term local control (LC) and survival after particle RT with minimal toxicity, while Combs et al. confirmed very low side effect rates and high LC in benign and primary settings, though outcomes were more limited in re-irradiated high-risk cases [13, 14].

Despite these advances, high-grade meningiomas continue to present significant therapeutic challenges. Consensus regarding optimal RT dose, target volume definition, and the relative benefits of photon versus particle-based approaches remains a subject of ongoing investigation and clinical trials [4, 10, 11]. Additionally, the molecular and genetic characteristics of these tumors are increasingly recognized as influential factors in prognosis and are being incorporated into modern treatment paradigms [15].

This study provides a comprehensive retrospective analysis of long-term outcomes and treatment-related toxicity in patients with intracranial WHO Grade II and III meningiomas treated with high-precision photon or particle radiotherapy at a single tertiary center, situating our findings within the latest advancements in the field.

## Materials and methods

### Study design and patient population

This retrospective cohort study evaluated patients with histologically confirmed WHO Grade II or III meningiomas who underwent RT in our department, between January 2006 and December 2023. The study was approved by the institutional ethics committee of our University and the requirement for written informed consent was waived due to the retrospective nature of data collection, in accordance with the ethical principles outlined in the Declaration of Helsinki.

All included patients were ≥ 15 years of age at the time of treatment and had a confirmed diagnosis of intermediate- or high-grade meningioma (WHO Grade II or III), according to the histopathological criteria outlined in the 2016 and updated 2021 World Health Organization Classification of Tumors of the Central Nervous System.

Tumor grading relied exclusively on histopathological assessment; molecular or cytogenetic markers (e.g., TERT promoter mutation, CDKN2A/B deletion, methylation class) were not systematically available and therefore not considered. Consequently, re-grading according to the WHO 2021 classification, which incorporates such molecular features, was not performed.

Diagnosis was made either at initial presentation or after recurrence and verified through neuropathological review at our institution. Patients were treated with either conventional photon RT or particle therapy using protons or carbon ions. To ensure sufficient clinical follow-up (FU) and assessment of outcomes, only patients with a minimum of two months of FU after completion of RT were included in the analysis.

Patients were excluded from the study if they had a known history of another intracranial malignancy, such as glioma or metastasis, or if clinical records and imaging data were incomplete. In addition, patients who were lost to FU or had insufficient clinical documentation within the first two months following RT were not included in the final cohort.

The indication for postoperative RT and the choice between photon and particle therapy were determined in an interdisciplinary neuro-oncological tumor board and, in selected cases, in a dedicated particle therapy tumor board. Clinical parameters (age, performance status), tumor-related features (grade, location, residual disease), and technical feasibility were considered when assigning treatment modality.

After application of these criteria, a total of 97 patients were eligible for analysis. The median age at diagnosis was 60.5 years (mean: 58.4 years; range: 15–88 years; standard deviation: ±15.2 years). The study population consisted of 48 male (49%) and 49 female (50.5%) patients. The majority of patients (*n* = 87; 89.7%) were diagnosed with WHO Grade II meningiomas, while 10 patients (10.3%) had Grade III tumors.

Extent of resection was determined based on surgical and radiological reports. Gross total resection (GTR) was defined as complete removal of all visible tumor, whereas subtotal resection (STR) indicated partial removal with residual tumor present. Among the 97 patients, 35 underwent GTR, 33 had STR, and in 29 cases, the extent of resection could not be determined due to missing documentation.

Based on anatomical location, tumors were classified as skull base (*n* = 30; 30.9%) or non-skull base (*n* = 67; 69.1%), with classification determined by neuroradiological assessment and surgical reports. Further demographic, pathological, and clinical characteristics of the study cohort are presented in Table 1. This cohort represents one of the largest single-institution experiences of radiotherapy in intermediate- and high-grade meningiomas, encompassing both photon- and particle-based treatment modalities over nearly two decades.

**Table 1:** Comprehensive summary

Category	Parameter	Value
Patients	Total number	97
	Sex (M / F)	48 (49.5%) / 49 (50.5%)
	Age (mean / median / range / SD)	58.3 / 60.5 yrs / 15–88 / ±15.2
	Histology (WHO II / III)	87 (89.7%) / 10 (10.3%)
	Tumor site (non-skull base / skull base)	67 (69.1%) / 30 (30.9%)
	Extent of resection (GTR / STR / Unknown)	35 (36.1%) / 33 (34.0%) / 29 (29.9%)
Radiation	Technique (single / fractionated)	0 (0%) / 97 (100%)
	Modality (photon / particle)	57 (58.2%) / 40 (41.8%)
	Dose (mean / median / range / SD)	58.7 / 59.4 Gy / 34–68 / ±5.3
	Dose groups (≤ 54 Gy / >54 Gy)	22 (22.7%) / 75 (77.3%)
Outcomes	OS (mean / median / range / SD)	63.1 / NR / 4–240 / ±57.2
	Median follow-up	41.0 mo
	PFS (mean / median / range)	41.6 / 32.0 mo / 3–210
	LC (mean / median / range)	44.1 / 33.0 mo / 4–240
	POF (mean / median / range)	16.3 / 0.0 mo / 0–204
	Survival (alive / deceased)	94 (96.9%) / 3 (3.1%)
	Overall progression (no / yes)	57 (58.8%) / 40 (41.2%)
	In-field progression (no / yes)	67 (69.1%) / 30 (30.9%)
	Out-field progression (no / yes)	75 (77.3%) / 22 (22.7%)
Early Toxicity (≤ 6 mo)	Alopecia	G1: 38, G2: 5, G3: 2
	Fatigue	G1: 24, G2: 8, G3: 2
	Headache	G1: 15, G3: 2
	Vertigo	G1: 12, G2: 2
	Nausea / Vomiting	G1: 6 / G1–2: 3
	Cephalgia / Singultus	G1: 4 / G1: 3
	Mental changes	G1: 1
Late Toxicity (> 6 mo)	Headache	G1: 12, G3: 2
	Seizures	G1: 9
	Vertigo / Hair loss	G1: 5, G2: 1 / G1: 4
	RICE	G1: 3, G2: 1, G3: 1
	Mental changes	0

Table 1 provides a detailed summary of patient demographics, treatment parameters, and oncologic outcomes, including WHO grade distribution, tumor location, radiation dose, and progression patterns

yrs: years, mo: months, SD: standard deviation, WHO: World Health Organization, Gy: Gray (unit of radiation dose), M / F: Male / Female, OS: Overall Survival, PFS: Progression-Free Survival, LC: Local Control, POF: Progression Outside Field, RICE: Radiation-induced cerebral contrast enhancement, G1, G2, G3: Grade I, II, III (toxicity grading according to CTCAE), CTCAE: Common Terminology, Criteria for Adverse Events, SRT: Stereotactic Radiotherapy, RT: Radiotherapy, NR: not reached

### Radiotherapy and treatment planning

Target volume delineation followed international consensus guidelines and institutional standards. The gross tumor volume (GTV) was defined as the contrast-enhancing lesion or postoperative residual tumor on MRI [16, 24]. The clinical target volume (CTV) was generated by adding a margin of 0.5–1.0 cm to the GTV to account for potential microscopic spread, with larger margins (up to 1–2 cm) applied in WHO grade II–III meningiomas depending on anatomical site and individual risk factors [14, 24]. The planning target volume (PTV) was created by expanding the CTV by 2–5 mm to compensate for setup uncertainties, immobilization accuracy, and institutional image-guided radiotherapy (IGRT) protocols [16, 17]. For skull base tumors, delineation adhered to ESTRO-ACROP consensus recommendations where applicable [16].

Treatment modality (photon vs. particle therapy) was selected through interdisciplinary tumor board discussion. Of the entire cohort (*n* = 97), 56 patients (57.7%) received photon radiotherapy and 41 patients (42.3%) underwent particle therapy, including 38 with protons (39.2%) and 3 with carbon ions (3.1%). No bimodal photon–particle regimens were used.

Photon radiotherapy was delivered with conformal, intensity-modulated, or IGRT techniques. The prescribed dose ranged from 34 to 68 Gy (mean 58.8 Gy; median 59.4 Gy; SD ± 6.5 Gy), in line with current recommendations [14, 17, 18, 24]. Proton and carbon ion therapy were prescribed at total doses of 45–64 Gy(RBE) (mean 58.6 Gy; median 59.4 Gy; SD ± 3.1 Gy), delivered in 1.8–3.0 Gy(RBE) fractions. Carbon ion therapy (*n* = 3) was administered using fractionated regimens according to institutional protocols. Dose prescriptions considered relative biological effectiveness (RBE) and linear energy transfer (LET) distribution, with optimization aimed at minimizing high-LET exposure to adjacent organs at risk [14, 18].

### Post-treatment follow-up

Following completion of RT, all patients underwent structured clinical and radiological FU, commencing approximately 12 weeks after the end of treatment. FU assessments included comprehensive neurological examinations and contrast-enhanced magnetic resonance imaging (MRI) of the brain. Imaging was performed at intervals of every 3 to 6 months during the first years after treatment, with extended intervals of up to 12 months thereafter, depending on tumor grade, anatomical location, and individual clinical course. The mean duration of radiological FU was 58.4 months (median: 37 months; range: 3–239 months), allowing for long-term evaluation of disease control and treatment-related sequelae. During FU, clinical endpoints such as tumor progression, local recurrence, and overall survival were systematically documented. Additionally, treatment-related toxicities, including radiation necrosis, were assessed and graded according to the Common Terminology Criteria for Adverse Events (CTCAE), version 5.0. Toxicities were categorized as early (occurring within six months post-radiotherapy) or late (beyond six months post-treatment).

Radiation-induced cerebral contrast enhancement (RICE) was diagnosed and classified according to the RANO working group criteria, based on MRI findings (contrast-enhancing T1-weighted lesions within the irradiated volume, accompanied by T2/FLAIR hyperintensity) and clinical presentation. Diagnostic adjudication was performed by a multidisciplinary tumor board including radiation oncologists, neuroradiologists, and neuro-oncologists.

### Outcome measures

The primary endpoints of this study were overall survival (OS), progression-free survival (PFS), and local control (LC). OS was defined as the duration from the initiation of RT to the date of death from any cause or the last FU date for surviving patients. PFS was measured from the start of RT to the first documented evidence of tumor progression, confirmed either radiologically or histologically. LC was defined as the time interval from RT initiation to the first occurrence of in-field tumor recurrence.

Treatment-related toxicity was evaluated as a secondary endpoint. Adverse events were systematically recorded and graded according to CTCAE version 5.0. Toxicities were further categorized based on their occurrence relative to the completion of RT.

### Statistical analysis

Survival curves for OS, PFS, and LC were generated using the Kaplan–Meier method. Time-to-event endpoints were estimated at 2-, 3-, 5-, and 8-year intervals to enable meaningful long-term outcome comparisons.

In the survival analysis, patients without an event at the time of last follow-up were censored. For OS, surviving patients were censored at the last follow-up. For PFS, patients without documented progression or who died from causes unrelated to meningioma were censored at the last follow-up date. For LC, patients without local recurrence at the last follow-up were censored. Censored observations are indicated by tick marks in the Kaplan–Meier plots.

Prognostic factors potentially associated with survival outcomes—including age at treatment, sex, WHO tumor grade, tumor location (skull base vs. non–skull base), radiotherapy dose, and treatment modality (photon vs. particle therapy)—were evaluated using univariate and multivariate Cox proportional hazards regression models. Hazard ratios (HR) with corresponding 95% confidence intervals (CI) were calculated, and a two-sided p-value < 0.05 was considered statistically significant.

Toxicity profiles were analyzed separately. Early toxicities (≤ 6 months) and late toxicities (> 6 months) were evaluated according to CTCAE version 5.0 criteria. Associations between treatment modality (photon vs. particle therapy) and specific toxicity endpoints were assessed using Spearman’s rank correlation analysis. Correlation coefficients (Spearman’s rho), 95% confidence intervals, and p-values were calculated, with *p* < 0.05 considered statistically significant.

All statistical analyses were performed using IBM SPSS Statistics, version 29.1 (IBM Corp., Armonk, NY, USA).

## Results

### Oncological outcomes

Radiotherapy was administered in a conventionally fractionated regimen to all patients (*n* = 97). Among these, 57 individuals (58.2%) received photon-based RT, whereas 40 patients (41.8%) were treated with particle therapy, either protons or carbon ions. The mean total prescribed dose across the cohort was 58.73 Gy (median: 59.4 Gy; SD: 5.3; range: 34–68 Gy).

At the time of last follow-up, 94 patients (96.9%) were alive and 3 patients (3.1%) had died. Median OS was not reached (NR), with a mean OS of 63.1 months (range: 4–240 months; SD ± 57.2). The median FU time, calculated using the reverse-Kaplan–Meier method, was 41 months.

The mean PFS was 41.6 months (median: 32.0 months), while the mean LC duration was 44.1 months (median: 33.0 months). Disease progression outside the irradiated field was observed with a mean latency of 16.3 months.

Overall, disease progression was documented in 40 patients (41.2%). Of these, 30 patients (30.9%) exhibited in-field recurrence, while 22 patients (22.7%) developed progression outside the irradiated volume. The remaining 57 patients (58.8%) remained free from progression throughout the FU period.

**Fig. 1:**
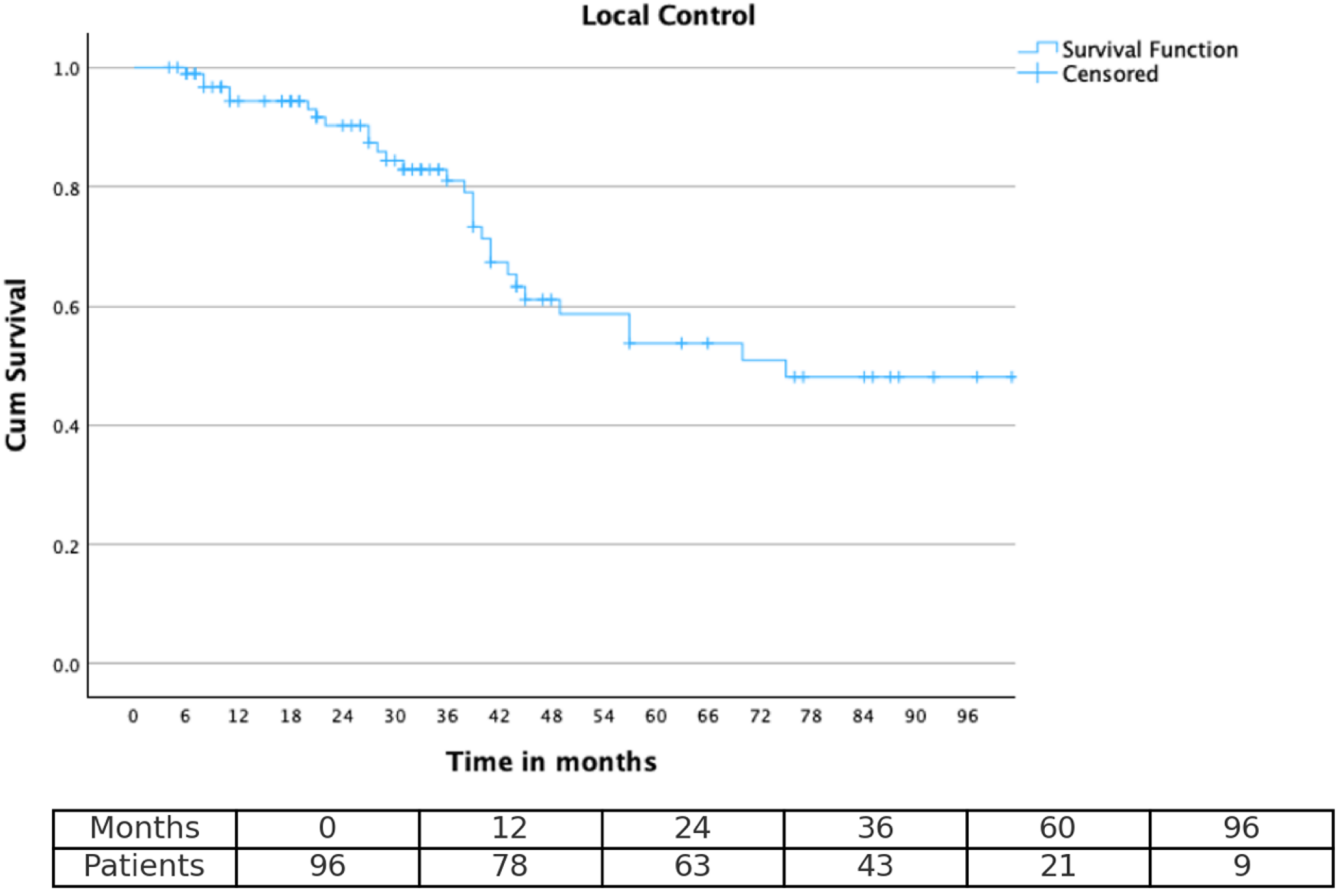
Illustrates the time-to-event analysis for local control (LC) across the cohort, showing 2-, 3-, 5-, and 8-year LC rates of 90.8%, 84.7%, 71.4%, and 69.4%, respectively

**Fig. 2:**
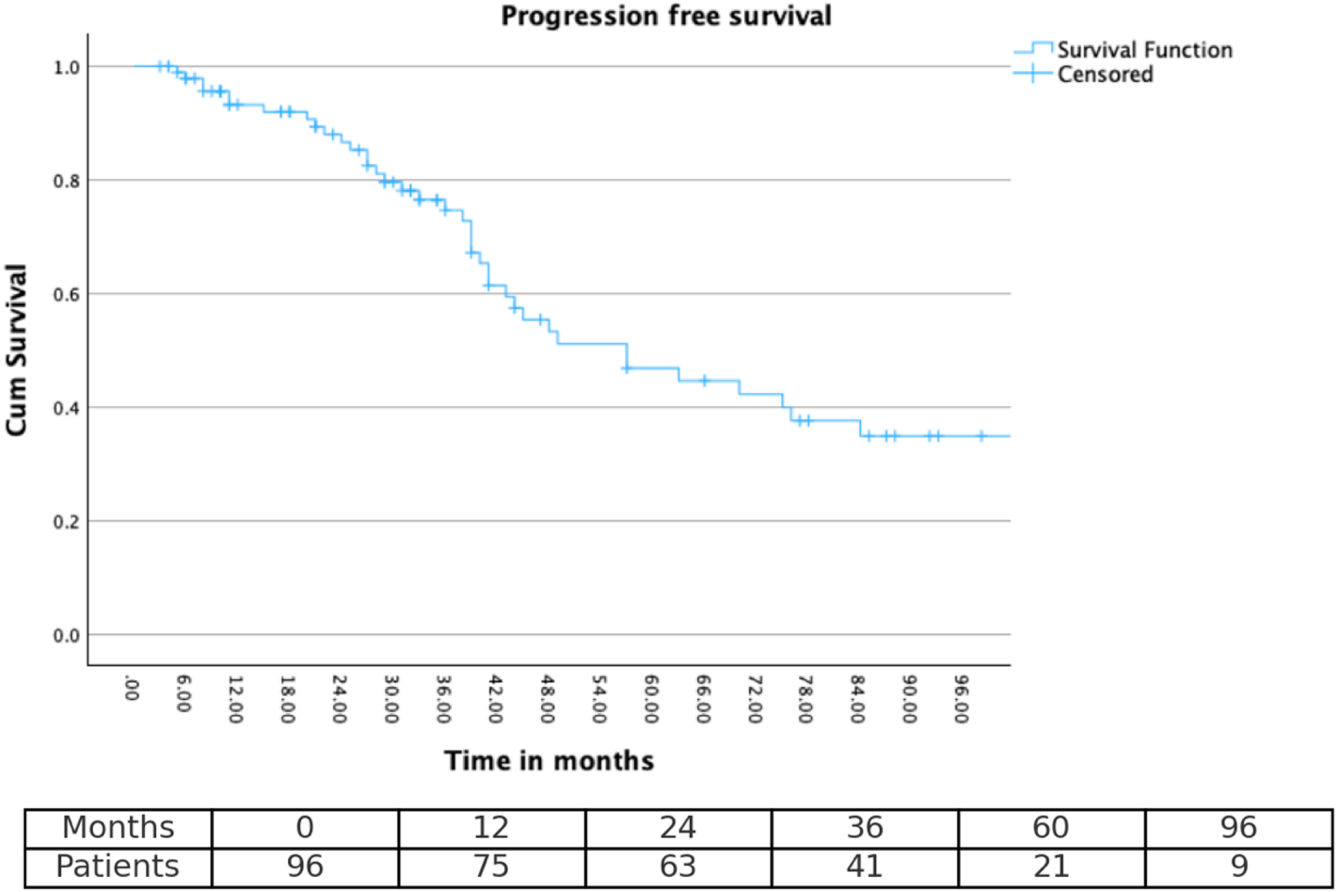
Displays Kaplan–Meier curves for progression-free survival (PFS), indicating sustained disease control over time, with PFS rates at 2, 3, 5, and 8 years of 87.8%, 79.6%, 65.0%, and 60.2%, respectively

**Fig. 3:**
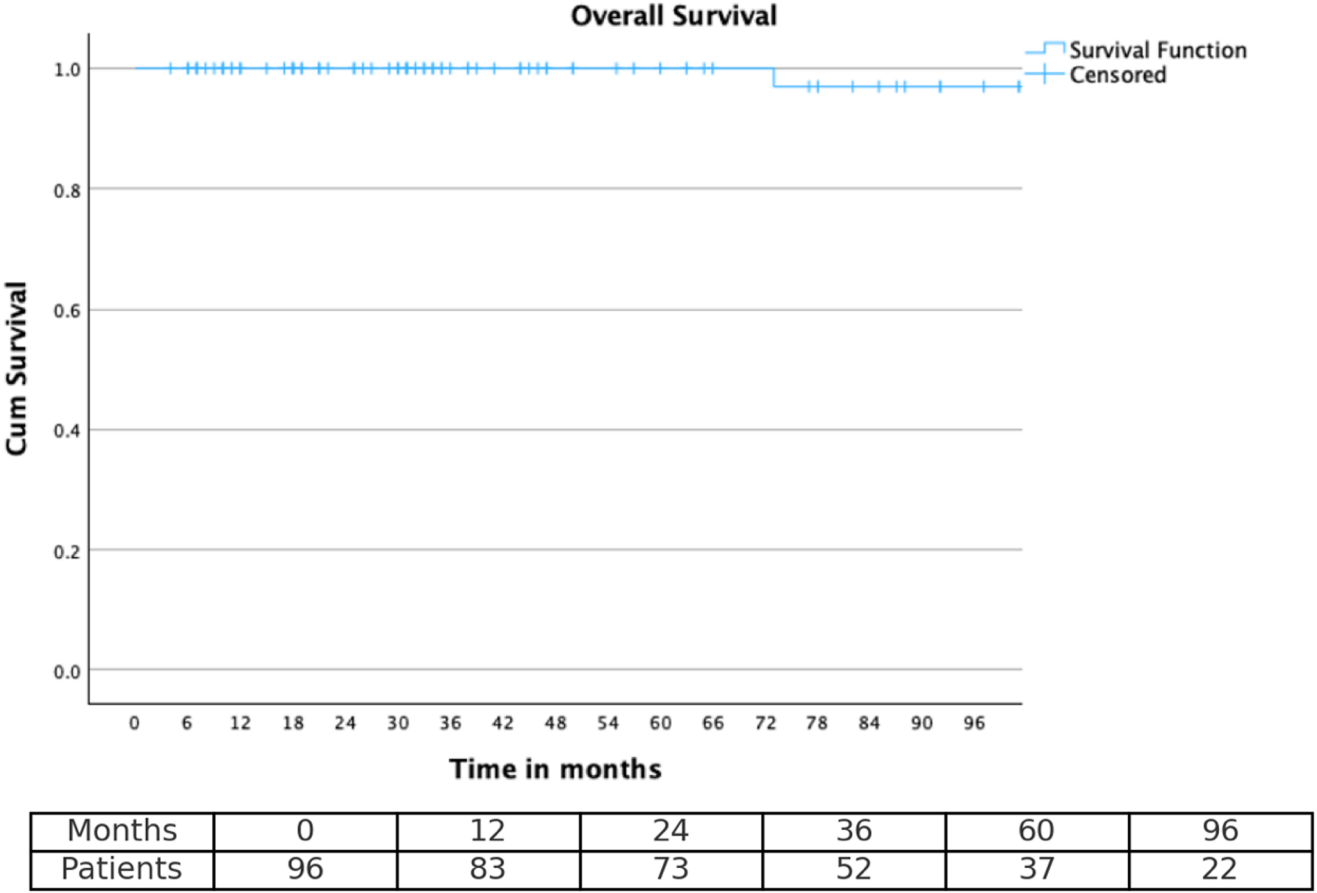
Depicts overall survival (OS), which remained exceptionally high in this cohort, with 2-, 3-, and 5-year OS rates at 100%, and 8-year OS at 99%

**Fig. 4:**
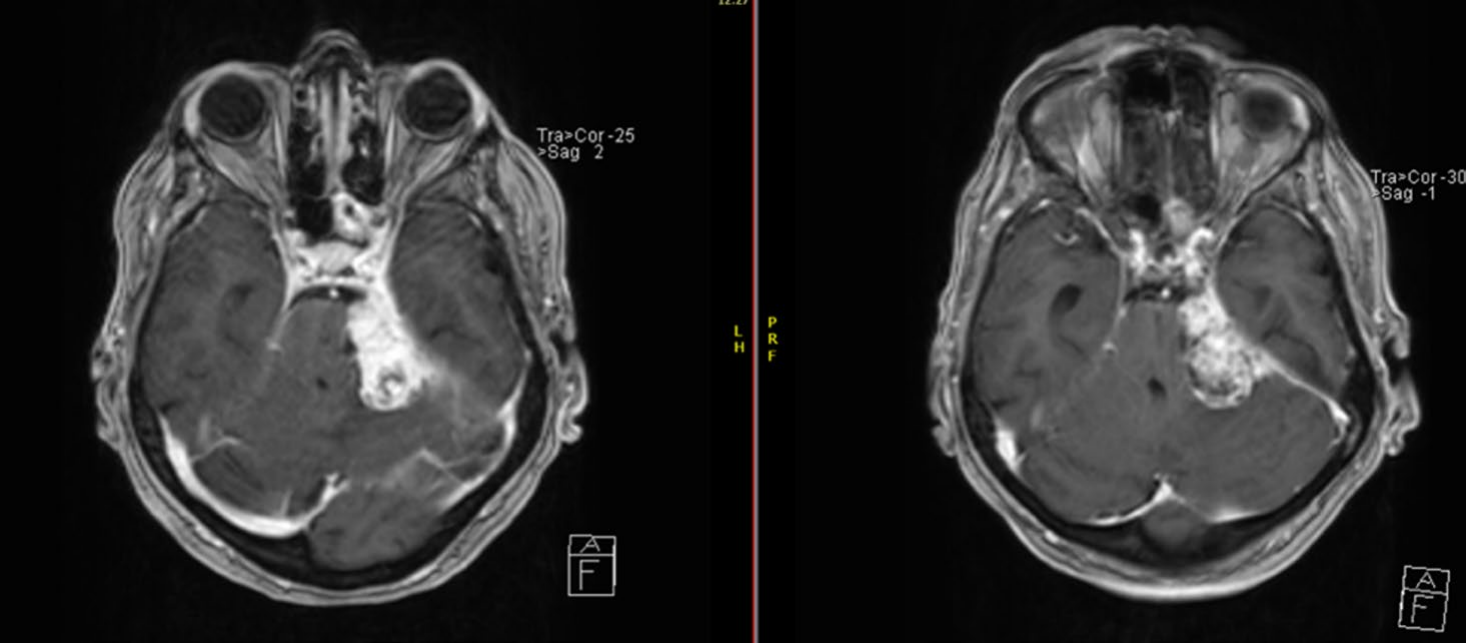
Axial T1-weighted contrast-enhanced MRI scans of a patient with a skull base meningioma before (left) and after (right) radiotherapy

Time-specific survival analysis demonstrated outcomes across all endpoints. At two years post-treatment, LC and PFS were 91.8% (8 events) and 88.7% (11 events), respectively, while overall survival remained at 100% (0 events). At three years, LC was 85.6% (14 events), PFS was 80.4% (19 events), and OS remained 100% (0 events). At five years, local control declined to 72.2% (27 events), PFS to 66.0% (33 events), although OS remained high at 100% (0 events). By eight years, LC and PFS further decreased to 69.1% (30 events) and 60.8% (38 events), respectively, with an OS of 99% (1 event). Kaplan–Meier curves for LC, PFS, and OS are shown in Figs. 1 and 2, and 3, while time-point-specific disease control and survival rates are summarized in Table 2. Representative axial MRI images before and after RT are presented in Fig. 4.

**Table 2:** Timepoint-based estimates

Timepoint	Local Control	Progression-free survival	Overall survival
2 years	91.8%	88.7%	100%
3 years	85.6%	80.4%	100%
5 years	72.2%	66.0%	100%
8 years	69.1%	60.8%	99%

Table 2 summarizes the 2-, 3-, 5-, and 8-year estimates of local control, progression-free survival, and overall survival

### Multivariate analysis

Cox proportional hazards regression was conducted to identify factors predictive of LC, PFS, and OS.

In the analysis of LC, none of the examined covariates reached statistical significance; however, there was a suggestive trend favoring higher radiation doses (> 54 Gy), which was associated with a reduced hazard of local recurrence (HR: 0.549, 95% CI: 0.258–1.164; *p* = 0.118). Other variables, including patient sex (HR: 0.583, 95% CI: 0.275–1.235; *p* = 0.159), WHO tumor grade (HR: 1.586, 95% CI: 0.603–4.171; *p* = 0.349), tumor location (HR: 0.708, 95% CI: 0.303–1.658; *p* = 0.427), and treatment modality (HR: 1.310, 95% CI: 0.556–3.087; *p* = 0.537) did not show significant associations.

For PFS, a similar non-significant trend toward improved outcomes with higher radiation dose was observed (HR: 0.654, 95% CI: 0.334–1.281; *p* = 0.216), though no variable reached statistical significance. Neither sex (HR: 0.772, 95% CI: 0.406–1.467; *p* = 0.429), WHO grade (HR: 1.763, 95% CI: 0.680–4.573; *p* = 0.243), nor tumor location (HR: 0.856, 95% CI: 0.423–1.733; *p* = 0.666) were significant predictors. Radiation type similarly did not affect outcome (HR: 1.435, 95% CI: 0.682–3.018; *p* = 0.341).

In the overall survival model, no independent predictors were identified. WHO Grade III tumors exhibited an elevated hazard ratio (HR: 4.664, 95% CI: 0.273–79.785; *p* = 0.288), although the wide confidence interval limits the robustness of interpretation. Similarly, sex (HR: 0.022, 95% CI: 0.000–2645.022; *p* = 0.521) and tumor location (HR: 1.414, 95% CI: 0.085–23.573; *p* = 0.809) demonstrated non-significant associations, reflecting the very low number of death events (*n* = 3).

A detailed summary of the univariable Cox regression analysis is provided in Table 3.

**Table 3:** Univariable cox regression results

Outcome	Variable	*p*-value	HR	95% CI (Lower – Upper)
Local Control	Sex (Male vs. Female)	0.159	0.583	0.275–1.235
	WHO Grade (II vs. III)	0.349	1.586	0.603–4.171
	Tumor Location (Non-skull base vs. Skull base)	0.427	0.708	0.303–1.658
	Radiation Dose (> 54 Gy vs. ≤54 Gy)	0.118	0.549	0.258–1.164
	Radiation Type (Photon vs. Particle)	0.537	1.310	0.556–3.087
Progression-Free Survival	Sex (Male vs. Female)	0.429	0.772	0.406–1.467
	WHO Grade (II vs. III)	0.243	1.763	0.680–4.573
	Tumor Location (Non-skull base vs. Skull base)	0.666	0.856	0.423–1.733
	Radiation Dose (> 54 Gy vs. ≤54 Gy)	0.216	0.654	0.334–1.281
	Radiation Type (Photon vs. Particle)	0.341	1.435	0.682–3.018

Table 3 reports the results of univariable analysis assessing potential predictors of LC, PFS, and OS, with hazard ratios and confidence intervals for sex, tumor grade, location, radiation dose, and modality

Note: Cox regression for OS was not performed due to the low number of events (*n* = 3)

HR = Hazard ratio, CI = confidence interval, p = p-value. HR reported for *p* < 0.05

### Treatment-related toxicity

According to CTCAE version 5.0, early treatment-related toxicities were predominantly mild to moderate. The most frequent acute event was alopecia (*n* = 45), mainly Grade I (*n* = 38), followed by Grade II (*n* = 5) and Grade III (*n* = 2). Fatigue was reported in 34 patients, mostly Grade I–II, and headache occurred in 17 patients (15 Grade I; 2 Grade III). Vertigo was observed in 14 patients (12 Grade I; 2 Grade II). Less frequent early adverse events included nausea (*n* = 6), vomiting (*n* = 3), cephalgia (*n* = 4), and hiccups (*n* = 3). Transient mental changes were rare (*n* = 1, Grade I).

Late toxicities were generally mild as well. Headache was documented in 14 patients (12 Grade I; 2 Grade III). Seizures occurred in 9 patients (all Grade I), and vertigo in 6 patients (5 Grade I; 1 Grade II). Alopecia persisted in 4 patients (all Grade I).

**Radiation-induced cerebral contrast enhancement (RICE)** was diagnosed in five patients:

- **Patient 1 (photon RT**,** 68 Gy)**: Severe motor and sensory aphasia due to progressive RICE in the right basotemporal region; no improvement under dexamethasone. The patient refused further diagnostic or therapeutic interventions.
- **Patient 2 (photon RT**,** 68 Gy)**: RICE with gait instability, vertigo, memory problems, depressive symptoms, and pre-existing mild word-finding difficulties.
- **Patient 3 (photon RT**,** 34 Gy**,** treatment discontinued due to epidural abscess)**: Developed fatigue and right arm motor dysfunction. MRI revealed partially regressive dural-based nodular leptomeningeal tumor components, alongside progressive leptomeningeal and intraparenchymal disease; features consistent with mixed progression and RICE.
- **Patient 4 (proton RT**,** 59 Gy)**: Radiogenic changes consistent with RICE, which later showed regression.
- **Patient 5 (proton RT**,** 60 Gy)**: MRI revealed findings suspicious for RICE associated with left-sided exophthalmos.

Severe late toxicity (Grade III) was rare, limited to two cases of headache and one case of RICE. No relevant late mental changes were observed. These findings underscore the overall safety of radiotherapy in this cohort, with low rates of severe adverse events and manageable treatment-related sequelae.

When stratified by treatment modality, no significant differences were observed in early toxicities (≤ 6 months post-RT) between photon and particle RT. In contrast, the analysis of late toxicities (≥ 6 months) demonstrated significant differences favoring particle RT. Specifically, vertigo (Spearman’s rho = − 0.256, *p* = 0.011, 95% CI = − 0.434 to − 0.051) and headache (Spearman’s rho = − 0.223, *p* = 0.028, 95% CI = − 0.404 to − 0.015) were significantly less frequent in patients treated with particle RT (Tables 1 and 4). These findings suggest a potential long-term benefit of particle RT in mitigating selected late toxicities.

**Table 4:** Toxicity comparison between photon and particle radiotherapy in meningioma

Toxicity parameter	Timepoint	*p*-value	Spearman’s rho (CC)	95% CI (Lower – Upper)
Hair loss	≤ 6 mo	0.845	–0.020	–0.219 to 0.181
	> 6 mo	0.180	0.137	–0.067 to 0.333
Nausea	≤ 6 mo	0.120	–0.159	–0.346 to 0.050
Vomiting	≤ 6 mo	0.245	0.119	–0.082 to 0.315
Vertigo	≤ 6 mo	0.613	–0.052	–0.250 to 0.149
	> 6 mo	0.011	–0.256	–0.434 to − 0.051
Cephalgia / Headache	≤ 6 mo	0.192	0.134	–0.067 to 0.329
	> 6 mo	0.028	–0.223	–0.404 to − 0.015
Mental change	≤ 6 mo	0.395	–0.087	–0.280 to 0.125
	> 6 mo	–	–	–
Fatigue	≤ 6 mo	0.734	–0.035	–0.230 to 0.165
RICE	≥ 6 mo	0.887	–0.015	–0.216 to 0.192
Seizure	≥ 6 mo	0.412	–0.084	–0.280 to 0.125

CC = Correlation Coefficient (Spearman’s rho), CI = confidence interval, p = p-value. Correlation is significant at the 0.05 level (2-tailed). RICE = Radiation-induced cerebral contrast enhancement. Early phase = ≤ 6 months post-RT; Late phase = > 6 months post-RT. Late mental change was not assessed

## Discussion

Recent advances in the understanding and treatment of WHO grade II and III meningiomas have informed evolving standards for RT, with multiple modern studies contextualizing the outcomes and toxicity seen in our cohort. Our study demonstrated excellent OS for grade II and III meningiomas receiving modern photon or particle RT, with OS rates exceeding 99% at 8 years, alongside encouraging LC and PFS rates. These findings align with recent phase II and retrospective studies that report 5-year PFS for grade II meningiomas in the range of 80–90% following adjuvant RT, as opposed to marked recurrence risk with surgery alone [9, 10, 19, 20].

In particular, the EORTC 22,042–26,042 phase II trial reported a 3-year PFS of 88.7% in patients with grade II meningiomas treated with adjuvant high-dose RT (60 Gy in 30 fractions), underscoring its efficacy [19]. Nevertheless, the study also observed grade 3–4 late toxicities in 8.9% of patients, emphasizing the importance of balancing disease control with treatment-related morbidity. Similarly, the RTOG 0539 trial, a risk-adapted phase II study, showed that adjuvant RT with 54 Gy yielded a 3-year PFS of 93.8% in patients with intermediate-risk (grade II) meningiomas following gross total resection [20]. In contrast, high-risk patients—including those with grade III disease, recurrence, or subtotal resection—received 60 Gy, achieving a 3-year PFS of 59.2% and an OS of 78.6%. These findings highlight both the therapeutic potential and the limitations of intensified RT in managing more aggressive tumor biology. Overall, our results add to the growing body of evidence supporting the role of modern RT in achieving durable disease control in grade II and III meningiomas, while also reinforcing the need for individualized treatment strategies to optimize efficacy and minimize toxicity.

However, not all data support a clear benefit of adjuvant RT. Yoon et al. reported in 158 patients with atypical meningiomas that median PFS was 59 months with RT versus 88 months without RT, and 5-year OS was 89% versus 83%, respectively. Their review of 22 additional studies showed highly divergent results, underscoring ongoing uncertainty and the need for randomized trials [21].

In our cohort, dose escalation above 54 Gy trended toward improved local control, echoing data from modern studies that advocate for consideration of higher doses or a carbon ion boost in selected high-risk patients [9–12, 19]. The MARCIE trial, for example, found a 3-year PFS of 80% for grade II lesions receiving a bimodal RT approach, with acceptable toxicity [22]. The prognosis for grade III meningiomas remains poor compared to grade II, despite aggressive surgery and RT. Our results of high recurrence rates and limited long-term survival are consonant with larger institutional series and meta-analyses, which report median survivals around 4 years and 5-year OS rates typically below 60% for grade III cases [10, 23]. Adjuvant RT is considered standard in view of the aggressive biology of high-grade meningiomas, with maximal safe debulking and early postoperative RT providing the best available outcomes, as supported by larger reviews and guideline statements [23, 24].

As demonstrated in large series of skull base meningiomas treated with FSRT or IMRT showing excellent LC and low toxicity [9, 12] and supported by reviews emphasizing the role of proton and carbon-ion therapy for dose escalation with conformal techniques [11], modern approaches increasingly improve outcomes in surgically challenging cases.

Similarly, Wu et al. conducted a systematic review of particle therapy in WHO grade II and III meningiomas, reporting local control rates ranging from 46.7% to 86% and overall survival rates from 0% to 100%, with better outcomes for atypical than for anaplastic tumors and a radiation necrosis incidence of 3.9% [25].

In the MARCIE trial combining carbon-ion boost with photon RT, 3-year local control reached 86.7% with acceptable toxicity [22], while other high-precision RT series reported 5-year local control rates of 55–80% depending on grade and treatment technique, with severe late toxicity remaining under 12% [9, 10].

Similarly, Sreenivasan et al. reported on 25 patients with 45 high-risk WHO grade II meningiomas treated postoperatively with stereotactic radiosurgery (SRS), demonstrating a 3-year LC rate of 84.6% and an OS of 96.0%. Notably, no local or marginal failures occurred in patients whose treatment targeted both the surgical cavity and residual disease or in those receiving hypofractionated SRS, further supporting the efficacy and safety of focused SRS approaches in selected high-risk cases [26].

Our findings of heterogeneous outcomes within WHO grade II and III cohorts are consistent with the growing recognition that histopathological grading alone is insufficient to capture the biological diversity of meningiomas. As emphasized by recent publications from the Heidelberg neuropathology group—key contributors to the 2021 WHO CNS tumor classification (Louis et al. 2021)—meningiomas demonstrate a broad spectrum of genetic alterations (e.g., NF2 inactivation, MEG3 and NDRG2 loss, Wnt/β-catenin and Hedgehog pathway involvement) and epigenetic changes. Notably, DNA methylation profiling enables more accurate risk stratification than histology alone, paving the way for future individualized radiotherapy approaches [27, 28].

This is further underscored by the recent International Consortium on Meningiomas (ICOM) consensus review, which highlights the integration of molecular biomarkers into grading and prognostication as a major future priority, although a unified taxonomy has not yet been established [29]. In addition to molecular biomarkers, advanced imaging modalities play an increasingly important role in modern radiotherapy planning. In particular, ^68Ga-DOTATOC PET has proven valuable for postoperative assessment and precise target volume delineation, and is regarded as an essential component of contemporary RT concepts [14, 24, 30]. Integration of DOTATOC PET into treatment planning may further enhance accuracy, minimize toxicity, and optimize outcomes.

Our data confirm that the majority of acute and late toxicities associated with modern radiotherapy are mild and reversible, with most patients experiencing only low-grade adverse events. Severe late toxicity (grade III) was observed in just 3 out of 98 patients, indicating a favorable safety profile. These findings are consistent with outcomes reported in contemporary clinical trials and large cohort studies.

For instance, in the prospective EORTC 22,042–26,042 trial, grade ≥ 3 late toxicities were reported in approximately 14% of patients receiving high-dose radiotherapy for atypical meningiomas [19]. Similarly, recent single-institution series report grade 3–4 acute and late toxicity rates ranging from 6% to 14%, with radionecrosis occurring in 8–18% of cases, largely depending on the dose delivered and the irradiated volume [9, 10, 12]. In the RTOG 0539 trial, most adverse events were grade 1 or 2 in severity. Importantly, only the high-risk cohort—which included patients with grade III, recurrent, or subtotally resected tumors—experienced serious complications, including a single case of fatal radionecrosis (grade 5 toxicity) [20].

Nonetheless, the risk of cognitive, ophthalmological, or neurologic sequelae remains a consideration, especially in the adjuvant setting and with dose escalation—requiring individualized risk–benefit analysis [9, 11, 12]. Our low observed rates of RICE and durable neurologic function reflect meticulous planning and the use of modern RT delivery, congruent with the trend in global reports [9, 11, 12].

Beyond toxicity, long-term patient-reported outcomes are also important. Fisher et al. reported that, despite comparable generic HRQOL to controls and convexity meningiomas, anterior/middle SBM patients had significantly better HRQOL than posterior SBM patients. Moreover, patients primarily treated with radiotherapy showed lower HRQOL in domains such as bodily pain and vitality compared to those undergoing surgery. These findings emphasize the need to consider not only disease control and toxicity but also functional and quality-of-life outcomes when tailoring treatment strategies [31].

Modern studies increasingly stress the need for refined risk stratification, including integration of molecular markers, to guide adjuvant RT and tailor dose and target volumes [11, 20, 23, 24]. Ongoing large-scale trials (e.g., NRG BN003) may clarify the necessity of adjuvant RT in gross-totally resected grade II tumors. Recently, molecular findings such as TERT promoter mutations, CDKN2A/B deletions, as well as chromosomal alterations like 1p loss and 1q gain have been identified as relevant predictors of recurrence, and are likely to be incorporated into future therapeutic decision-making [11, 23, 32, 33].

Such integration of molecular, clinical, and imaging features represents a central recommendation of the ICOM consensus, providing a framework for future personalized management of meningiomas [29].

As with other retrospective and single-institution studies, heterogeneity in surgery, histologic review, RT technique, and imaging follow-up limit direct comparison. Nevertheless, the parallels between our real-world experience and major contemporary prospective trials underscore the robustness and clinical relevance of our findings.

A detailed summary of these key studies, including population, RT dose/technique, outcomes, and toxicity findings, is presented in Table 5 below.

**Table 5:** Key studies on adjuvant radiotherapy for WHO grade II–III meningiomas

Study / Series	Population / Design	RT Dose / Technique	PFS / LC (%)	Key Toxicity / QoL Findings
Wiemels et al. (Particle therapy) [2]	Grade II/III, retrospective/prospective	Carbon/proton	3–5 y LC: up to 80	< 12% late toxicity
EORTC 22,042–26,042 [19]	Grade II, prospective	60 Gy, 3D-CRT	3-y PFS: 88.7	14% grade 3–4 late events
RTOG 0539 [20]	Phase II, risk stratified	54–60 Gy, IMRT	3-y PFS: 94 / 59 (int./hi risk)	Low severe toxicity
MARCIE trial [21, 22]	Grade II, phase II	Photon + carbon-ion	3-y PFS: 80	Mostly mild; 1 fatal necrosis
Krcek et al. [34]	Grade I–III, retrospective (*n* = 200)	PBS Proton	5-y OS: 95.7 (G1), 81.8 (G2/3)	Very low high-grade toxicity; stable QoL
Lisowski et al. [35]	Grade I–III, retrospective (*n* = 119)	Photon RT (FSRT/IMRT)	5-y LC: 82, 10-y LC: 73	HRQoL ↓ in some domains; 4.2% severe late toxicity
Kent et al. [36]	Grade II/III, retrospective (*n* = 66)	Surgery ± RT (54–60 Gy)	Median PFS 3.2 y, OS 8.8 y	Adjuvant RT ↑ PFS (HR 0.36); Ki-67 > 10% → worse PFS
Present study	Grade II/III, retrospective	34–68 Gy, photon/particle	5-y LC: 72.2, PFS: 66, 8-y OS: 99	3% severe late toxicity

Table 5 compares major clinical studies on radiotherapy in WHO Grade II–III meningiomas, including design, dose, technique, outcomes, and toxicity findings

RT: Radiotherapy, PFS: Progression-Free Survival, LC: Local Control, QoL: Quality of Life, OS: Overall Survival, 3D-CRT: Three-Dimensional Conformal Radiotherapy, IMRT: Intensity-Modulated Radiotherapy, FSRT: Fractionated Stereotactic Radiotherapy, PBS: Pencil Beam Scanning (proton therapy technique), HRQoL: Health-Related Quality of Life, HR: Hazard Ratio

## Conclusion

Our findings demonstrate that modern photon and particle radiotherapy is an effective and safe approach for achieving long-term disease control in high-grade meningiomas. At eight years of follow-up, overall survival exceeded 99%, with local control and progression-free survival remaining at encouraging levels. Dose escalation above 54 Gy showed a trend toward improved local control, and severe late toxicities were exceedingly rare; most treatment-related side effects were low-grade and reversible.

Taken together, these data reaffirm modern radiotherapy as a key component of multimodal treatment for high-grade meningiomas. In the future, the integration of molecular and genetic markers (e.g., TERT promoter mutations, CDKN2A/B deletions) into risk stratification, along with technological advances and large-scale prospective validation studies, will play a pivotal role in defining the most appropriate and personalized treatment strategies for this challenging tumor subtype.

## Data Availability

No datasets were generated or analysed during the current study.
